# 

**Published:** 1905-07

**Authors:** 


### Index to New Series Volume XLIV.

(Whole Number Volume LX.)

AUGUST, 1904,—JULY, 1905.

PAGE.

ABDOMEN, Pendulous..........643
Abdominal Operations, Treatment of Patients Undergoing..... 1
Abdominal Section, Preparation of Patient for and Treatment of.....102
Acetezone in Summer Diarrhea.....117
Acne of the Face.................173
Albuminuria, Glycogen in Diabetic.. 37
Alexander Operation, Immediate and Remote Results...................374
Anesthetic, Best for Children.......112
Aneurism, Report of an Abdominal. 18
Angioma Hypertrophicum..........566
Antitetanic Serum as a Prophylactic. 41
Antonius, Appeal of, Rejected....... 53
Apenta Water in Constipation and Obesity ......................... 37
Apollinaris, Facts about............116
Unrivaled ...........823
Appendicitis ......................385
Concerning the Etiology of ..............773
Perilous Calms of......580
Appetite, for a Failing..............309
Association Invites Society..........255
Athletics in Professional Colleges...435

BAINBRIDGE, WILLIAM S., (a) Multiple Hernia; (b) Angioma Hypertrophicum............566
Baly Medal Award ................823
Bayliss, A. W., Treatment of Disease by Means Other than Drugs.802
Bentz, Charles A., Traumatic Tetanus.633
Benzoin, Therapeutic Use of........ 33
Beta-Eucain vs. Cocain.............398
Lactate of............672
Bishop, Mrs. J. F., Chinese Doctors and Medical Treatment...........143
Bladder, Irritability of.............396
Books Received............70, 141, 203, 271, 344, 416, 488, 559, 630, 698, 770

PAGE.

Bubo, Abortion of..................164
Burrows, Lorenzo, Practical Use of Ophthalmometer .................512
Busch, Louisa, Arrest of............615
Louisa, Conviction of.......763

CALOX, a New Dentifrice........325
Cardiac Diseases, Essential Medication in .......................426
Cardiac Diseases, Essential Medication in ..........................426
Celloidin in Surgery...............466
Cerebrospinal Meningitis ...........457
Cervix, Consequences of Lacerations of ......................229
Gonorrhea of...............164
Chapin, Henry Dwight, Practice of Infant Feeding ..................794
Chinese Doctors and Medical Treatment ....................143
Management of Transverse Presentation .............241
Medicine and Surgery in New York...............153
Clinton, Marshall, Abdominal Aneurism ..................... 18
Marshall, Analysis of Fifty Herniotomies ............ 92
Marshall, Infected Wounds of Extremities............284
Collargolum Enemata...............112
on Bacteria of Tubercle Bacillus ............397

**College and Hospital Notes:**

Bacteriology, Clinical Course, U. of B........................552
Batavia Hospital................477
Brigham Hall, Canandaigua.....477
Buffalo General Hospital........ 61

PAGE.

Buffalo Hospital Sisters of Charity .......................... 61
Buffalo State Hospital .........827
Detroit College of Medicine .....690
Hackley Hospital, Muskegon, Mich. .......................329
Lincoln Hospital, New York....412
Manhattan Eye, Ear and Throat Hospital ....................477
McGill University, Montreal....690
Medical Department, University of Buffalo.......132, 329, 620, 688
Mercy Hospital, Buffalo.132, 198, 260
Michigan College of Medicine and Surgery.................412
New York Post-Graduate Hospital and Medical School........329
New York School of Clinical Medicine .................... 62
New York Skin and Cancer Hospital ...............261, 552, 620
Niagara Hospital, Buffalo.......261
Providence Retreat.............690
Punton Sanitarium, Kansas City.133
Quarantine Hospital............ 62
Saint Bartholomew's Hospital, London .....................691
Saint Luke's Hospital, Utica....261
State Hospital for Incipient Tuberculosis .................... 61
Syracuse University, College of Medicine ....................690
Texas Tuberculosis Sanitarium..198
University of Buffalo Lectures...477
University Day, U. of B.........552

Consolidation of Society and Association ............................ 54
Consumption in Germany........... 53
Consumptives, Sanatorium for......514
Convalescents, Considerations Relating to ........................792

**Correspondence:**

American Medical Association Council on Pharmacy—George H. Simmons ...............676
American Medical Association at Portland—William House.....673
American Society for Study of Alcohol, etc.—T. B. Crothers..119
Complimentary Dinner to John B. Chapin—Edward N. Brush....180
Klotz's Case of Syphilitic Reinfection—A. D. Mewborn......244
Medical Department of Grosvenor Library—Edward P. Van Duzee .........................243

PAGE.

Walter Reed Memorial Association—C. DeWitt.............. 42

Cott, George F., Acknowledgment to.763
George F., Middle-ear Suppuration .......................572
Cough in Children..................308
Creveling, J. P., Pendulous Abdomen.643
Cumston, Charles G., Metrorrhagia in Interstitial Nephritis...........718
Current of High Voltage, Effects of.788
Cystitis ............................286

DAVIS, WILLIAM E. B., Statue of .........................189
Diabetes, Mellitus..................648
Diarrhea, Bacillus of Dysentery in.. 22
Diarrheas in Infancy...........119, 308
Dietetics, Problems in...............303
Diphtheria, Vitality of Germs of.... 54
Dorsett, Walter B. Abdominal Section ...........................102

EAR, Grain of Rice in............324
Eczema, Gouty................173
Education, Council of A. M. A......691
Empyemas of Accessory Cavities in Children ......................444
Endermol, a New Vehicle for Ointments .........................168
Epididymitis, Treatment of.........165
Epilepsy, Horse Nettle in...........309
Eucain and Eucain-Adrenalin for Local Anesthesia.............171
Lactate .....................171
Euphthalmin in Ophthalmic Examination ........................222
Eye Work, Electro-Magnet in........515

FAIRCHILD Scholarship.........404
Fakir, Pure and Simple........461
Fibroid Tumor Complicating Gestation ............................452
Fibrosis, Circumscribed.............162
Finch, Isaac A., Conviction of.......615
Fireworks Ordinance ..............823
Flannelette, Inflammable ...........807
Food Adulteration.................. 87
Frazee, A. B., Common Forms of Headache ......................223

GALACTAGOG, an Efficient.....172
Gallbladder, Rupture of.......596
Garbage System in Paris............187
Gastric Ulcers, Classification of......300

PAGE.
Gauze Drains .....................541
Gloves, Substitute for Rubber.......399
Goitre, a Case of Cystic...........561
Grant, John H., Adulteration of Food 87
Grosvenor, J. W., Postpartum Hemorrhage ........................ 73
Gonococcus, Longevity of the.......165
Gonorrheal Infection, General.......165
Glycothymoline in Nasal Catarrh....396
An Oronasal and General Antiseptic................671

HANLEY, L. G., Acute Hemorrhagic Pancreatitis...........293
Hanley, Operation for Ptosis of Kidney ...........................389
Hair, to Prevent Falling...........461
Hats, Inconvenience of.............115
Headache, Common Forms of.......223
Healer, a New at Dunkirk..........404
Hernia, Radical Cure of Inguinal.... 95
Multiple ..................566
Herniotomies, Analysis of Fifty..... 92
Home, a Letter from................683
Hospitals in New York City........ 53
Houston, Sam, Methods and Purposes of Pension Examinations... 12
Humerus, Fracture of..............385
Hysterectomy, Abdominal vs. Vaginal 239

ICTERUS from Insect Bites and Gallbladder Disease ........... 38

**Illustrations:**

Bainbridge, Angioma Hypertrophicum:
Fig. 1, Before First Operation.569
Fig, 2, Before Second Operation .................570
Fig, 3, Present Condition.....571
Bainbridge, Multiple Hernia.....567
Bryant, Joseph D., Portrait......545
Clinton, Abdominal Aneurism (Tumor) .................... 20
Clinton, Gluteal Lipoma and Inguinal Hernia ................ 93
Hardon, Foreign Bodies in Appendix ........................583
Johnston, Osteomyelitis:
Fig. 1, After Operation......350
Fig. 2, Skiagraph ...........351
Fig. 3, After Recovery.......353
Fig. 4, Skiagraph ...........353
Fig. 5, Anterior View, Seven Years After Exsection ...............354

PAGE.
Fig. 6, Skiagraph ...........355
Fig. 7, After Recovery.......357
Fig. 8, After Recovery.......358
Fig. 9, Eight Years After Operation ............359
Fig. 10, Skiagraph ...........360
McMurtry, L. S., Portrait......821
O'Brien, E. C. W., Portrait.....618
Park, Foreign Bodies in Pharynx, etc.:
Fig. 1, Radiograph ..........450
Fig. 2, Pin Removed from Child's Throat ......451
Ruggles, Genitourinary Table:
Fig. 1, Table Level..........518
Fig. 2, Pelvis Elevated........518
Fig. 3, For Urethroscopic Examinations ............519
Fig. 4, Trendelenberg Posture.520
Thompson, Fibroid and Pregnancy:
Fig. 1, Anterior View........453
Fig. 2, Posterior View.......454

Infant Feeding, Practice of.........794
Mortality, Prevention of.... 25
The Premature..............653
Toilet of Newborn..........240
Infantile Colic ..................... 27
Gastritis .................. 27
Infection, Flowers and Birds as Bearers of......................682
Infections of the Newborn..........109
Insane, Care of....................275
Insanity, Toxemia and Infections as Causes .........................362
Isthmian Canal Commission Reorganised ............................683

**Items:**

American Medical Association, Rates to Portland............771
Antikamnia Chemical Co., Calendar .........................490
Apollinaris, World's Fair Grand Prize .......................490
Arlington Chemical Co., Sketches346
Arlington Chemical Co., Law and the Doctor, Vol. II...........490
Battle & Co., Intestinal Parasites, .............72, 274, 418, 632
Breitenbach, M. J. & Co., Daily Memorandum, 1905...........418
Civil Service Examinations.....142
Davis Pharmaceutical Co.......274
Denver Chemical Co., Booklet on Antiphlogistine...........560, 632
Dios Chemical Co., Visiting List, 1905 ..........................346

PAGE.
Hudor Company................490
Lactopeptine Calendar.......... 72
Lambert Pharmacal Co., Calendar ..........................490
Marchand's Safety Valve Stopper ..........................418
Mellier Drug Co., Calendar.....490
Mellin's Food Co., World's Fair Grand Prize .................418
New York Pharmacal Association, Haeckel's Chart.........632
Pepto-Mangan in Treatment of Hookworm Anemia ..........828
Philippine Exhibit at Saint Louis Fair ........................ 71
Pope Bicycle Daily Calendar, 1905 .........................418
President Roosevelt at World's Fair ..........................346
Subscriber (The), and the Law.206
Taylor, Physicians' Account Book.345
Wabash Railroad...............142
Warner, William R. & Co.,
World's Fair Catalogue......... 72
World's Fair Prize............345

JACKSON Health Resort Nurses' Commencement ...............823
Jacobson, Nathan, Cystic Goitre....561
Jardin, Milo F., Arrest of...........615
Johnston, George Ben, Removal of Shaft of Tibia for Osteomyelitis...347

KIDNEY, Operation for Ptosis of.389
King, James E., Immediate and Remote Results of Alexander Operation .........................374
Krauss, William C., Medical Library in Buffalo.......................499

LAVAGE in Children........... 24
Legislative Bills, Fine Triad of ..............................550

**Leading Articles:**

Advertisers and Advertising....677
Alumni Association, Medical Department U. of B...........808
American Association of Obstetricians and Gynecologists.....185
American Gynecological Society.816
American Journal of Urology...253
American Medical Association Meeting at Portland........ .......323, 545, 614, 680, 762, 821
PAGE.
Carbolic Acid, A Fight Against.254
Dust and Noise as Causes of Disease .......................468
Exhibit of Historic Interest.....251
Isthmian Canal Problem........612
Medical Record, Editor of Retires ........................123
Medical Society State of N. Y...543
Millican, Kenneth W...........186
"New York Standard"..........125
Osteopaths and the State.......546
Physicians' Hostelry at World's Fair ........................124
Reciprocity in Licensure........679
Rush, Benjamin................ 49
Substitution, Another Conviction for .........................471
Substitution, English Courts Deal With ....................... 51
Tuberculosis, How to Limit Spread of....................249
Tuberculosis, Prevention of Pulmonary .....................402
University of Buffalo Endowments ........................ 47
University of Buffalo Standard Advanced ..................760
University and Hospital Bulletin.121
War, Medical Aspects of the Eastern ....................182
Water Supply of Buffalo........122
Weigel, Dr. Louis A...........250

Legume Flour in Atrophic Infants...455
Le Seur, John W., Professional Esprit de Corps.....................577
Lewis, F. Park, The Graduate Nurse.781
Library, Medical in Buffalo.........499

**Literary Notes:**

Addresses at Dinner to Dr. Roosa ...................... 70
American Journal of Nursing...417
American Journal of Orthopedic Surgery ....................345
American Journal of Surgery...632
Annals of Surgery............417
Appleton, D. & Co., Die Deutsche Klinik ......................699
Blakiston, P. Son & Co., Catalogue ........................344
Blakiston, P. Son & Co., Exhibit at Atlantic City............. 71
Georgia Practician.............560
Henry Phipps Institute Report..700
Iowa Medical Journal..........560
Iris, Vol. VIII., 1905..........700
Journal of Obstetrics and Gynecology of the British Empire..274

PAGE.
King's Medical Prescriptions....560
Lea Bros. & Co., General Catalogue ....418
Lea Bros. & Co., Gray's Anatomy ....828
Lippincott, J. B. Co., Krehl's Pathology ....632
Literary Digest ....345
Macmillan Co., Notes for Guidance of Authors....700
Medical Book News....142
Medical Notes and Queries....700
New York State Hospital for Ruptured Children....700
Old Dominion Journal of Medicine ....345
Saint Louis Courier of Medicine, Care of Premature Infants....489
Southern Medicine and Gaillard's Medical Journal....274
Surgery, Gynecology, and Obstetrics ....771
Western Medical Review.... 71
Wood, William & Co., Brochure (100 Yrs. Med. Pub.)....489

Long, Eli H., Medication in Cardiac Diseases ....426
Lumbago, For....308
Lymphaticus, Status and Sudden Death ....230
Lynde, Samuel H., Memorial....533
Lytle, Albert T., The Nurse or the Doctor? ....798

MACLEOD, J. A., Treatment of Patients Undergoing Abdominal Operations.... 1
McGuire, Edgar R., Status Lymphaticus; Relation to Sudden Death ....230
F. W., Fibroma of the Ovary ....294
McKee, T. J., Physician in the Making ....207
Malaria, Campaign Against....571
For ....396
Malpractice Suits....188
Measles, Treatment of.... 25
Medical Research, Institute of....188
Medicine, Accomplishing Today....491
Antient Cymric ....255
Definition of Practice of..549
Medicines, Sale of Proprietary....188
Menstrual Disorders, Treatment of.. 40
Mexico, Notes of Travel in....377
Middle-Ear Suppuration....572
Milk, Certified....405

PAGE.
Millener, F. H., Effects of Alternating Current of High Voltage....788
Morris, Robert T., Peritoneal Adhesions, etc....385
Mouth, Hygiene of....614
Myers, Reuben S., Memorial....531

NEPHRITIS, Metrorrhagia in Interstitial ....718
Nephritis, Surgery of....586
Nervous Excitement and Neuralgia..156
Nipples, Excoriated.... 94
Nurse or Doctor ....798
The Graduate....781

**Obituary:**

Allingham, Herbert William....410
Allison, Henry E....410
Boyle, Julius J....825
Chambers, Jacob....191
Coon, Mortimer S....617
Crisfield, James E....551
Dornan, William J....617
Finsen, Niels R....191
Gillette, George W....409
Gleason, Rachel Brooks....617
Greene, Cordelia A....551
Helmer, Josiah H....327
Hysell, James H....825
Kittinger, Martin S....190
Lyman, Henry Munson....409
Lynde, S. H....257
Manley, Thomas H....474
Martin, Eugene Edward....326
Mastin, Charlotte E....825
Myers, R. S....257
O'Brien, E. C. W....618
O'Hara, Arthur T....474
Palmer, George M....825
Palmer, William E....408
Pearce, Frank Savary.... 57
Pennington, Byron C....474
Preston, W. D....551
Price, Mordecai....327, 407
Pryor, William Rice....191
Rose, Madison H....474
Seymour, William Wotkyns....256
Showerman, B. F....826
Smart, Col. Charles....686
Smith, Hamilton E....409
Smith, Ira P....826
Snetsinger, Josephine....686
Strong, Walter D. O. K....551
Whitcomb, Frank W.... 57
Williams, Julian Taintor....686
Wise, Henry A.... 57
Young, Edson H....551

PAGE.

O'BRIEN, E. C. W., Memorial...591
O'Hara, A. T., Memorial.....534
Ophthalmia, New Prophylaxis and Cure for Gonorrheal.............712
Ophthalmometer, Practical Use of..512
Osteomyelitis of Shaft of Tibia.....347
Osteopath Fined....................616
Bill Defeated............681
Bill in Pennsylvania......684
Otitis Media in Infancy............. 26
Media, Treatment of.........720
Ovary, Fibroma of.................294

PANAMA, Trained Nurses for...477
Panzer, B., Empyemas of Accessory Cavities.....................444
Pancreatitis, Acute Hemorrhagic....293
Paralysis, Transposition of Hamstring Tendons in................ 24
Paranoia as it Relates to Homicide..419
Pension Examinations, Methods and Purposes of...................... 12
Pericarditis, Tuberculous........... 35
Peritoneal Adhesions..............385

**Personal.**

Brewer, George E..............291
Bryant, Joseph D.; Tate, Magnus A.; Miller, Jacob.........617
Burke, Joseph; Deaver, John B.; Himmelsbach, G. A.; Hussey, E. P.; Haase, Charles; McMurtry, L. S.; Potter, Irving, W.; Stockton, Charles G..... 56
Chase, Walter B.; Davis, Thomas D.; Dudley, E. C.; Hinkel, F. Whitehill; Moyer, Harold N.; Murphy, John B.; Runyan, Joseph P.; Renner, W. Scott. 55
Clark, Edward; Smith, Eugene; Panzer, Bernard ............128
Clinton, Marshall; Jewett, Charles Sherman; Grove, Benjamin H.; Kenerson, Vertner; Carr, Francis J.; Park, Roswell; McMurtry, L. S.............472
Crockett, M. A.; Keyes, Regina Flood ......................261
Dudley, E. C.; Durand, Charles F.; Greene, Dewitt C.; Hubbell, A. A.; Dowd, J. Henry; Barker, Lewellys F.; Buswell, Henry C.; Hill, Henry W.; Marcy, William H...........685
Grant, John H.; Millener, Frederick H.; Pohlman, Augustus G.; Wilgus, Sidney D........127
PAGE.
Hanley, L. G.; Meyer, E. J.; Simpson, Burton T.; Wright, A. B.........................238
Harrington, D. W.; Cott, George F.; Briggs, A. H.; Gallagher, James L.; Pryor, John H.; Walker, Edwin...............473
Hicks, W. Scott; Pratt, John R.; Briggs, A. H.; Wells, Brooks H.; Roberts, John B.; Dudley, E. C.; Ruf, Frank A..........256
Kelly, Catherine E.; Gibson, James A.; Clark, L. Pierce; Breuer, Max C.; Shurly, E. L.; Otto, Jacob S.; Lewis, F. Park; Porter, Eugene H.; Hill, F. E.; Greene, Joel H...764
Low, Frank W.; Smith, Eugene; Bovée, J. Wesley.............190
Morris, Robert T.; Winn, John F.; Fronzcak, Francis E.; Grove, Benjamin H..........825
Murphy, John B.; Griffith, J. D.; Stranahan, C. W.; Lewis, F. Park; Bandler, Samuel Wyllis ..........................824
Putnam, James Wright; Gipple, F. M.; Jayne, A. W.; Foster, Frank P.; North, Charles H.; Congdon, Charles E.; Greene, Walter D....................406
Reed, Charles A. L.; O'Brien, E. C. W.; Ring, William G.; Bessemer, Martin; Merck, Willy; Wilcox, Dewitt G........550
Renner, W. Scott; Millener, F. H.; Dignen, W. E.; Barrett, F. J.; Bradbury, A. R.; Jelks, F. W., and James T.........326
Ricketts, Edwin; Crile, George W. ..........................407
Sinclair, William Japp; Hendee, Lawrence ....................325
Vander Veer, Albert; Ullman, Julius; Beebe, G. E.; Cushny, A. R.; Buswell, H. C.; Banta, Charles W...................616
Weed, Harry M.; Kenerson, Vertner .....................765
Wende, Grover W.; Stockton, Charles G. ..................189
Williams, Herbert U............236

Petrogen, Therapeutic Value of.....467
Pharynx and Esophagus, Foreign Bodies in........................449
Phillips, William L., New Cure of Gonorrheal Ophthalmia...........712
Physical Signs in Children..........657
Physician in the Making............207

PAGE.
Physicians, Mortality among........780
Placenta Previa, Cesarean Section for ................................312
Pneumonia and Pleurisy Simulating Appendicitis ......... 23
Treatment of............536
Polyclinic, For Sick Poor...........530
Postpartum Hemorrhage............ 73
Practice of Medicine, Definition of..684
Pregnancy, Vomiting of............181
Professional Esprit de Corps........577

**Progress in Medical Science.**

Genitourinary and Syphilitic Diseases, by Nelson W. Wilson..162
Medicine, by Nelson W. Wilson. 33
Pediatrics, by Maud J. Frye.... ................22, 109, 655
Surgery, Obstetrics and Gynecology, by William Warren Potter ...................27, 239
Therapeutic Notes, by Nelson W. Wilson .................308, 395

Puerperal Sepsis, Prophylaxis of.... 29
Putnam, James Wright, Paranoia as Related to Homicide..............419
Pyramidon, Salts of................809

QUARANTINE ................646

RECTAL Examination in Children ..........................656
Red Cross, New Charter for........542

**Reviews.**

Allen: Radio and Phototherapy.262
Bailey: Textbook of Histology..623
Ball: Modern Ophthalmology..263
Ball: Essentials of Bacteriology.556
Barker: Human Anatomy......628
Bartley: Physiologic and Clinical Chemistry ...................555
Beck: Röntgen Ray Diagnosis and Therapy..................138
v. Bergmann: Practical Surgery, Vol. II., 138; Vol. III., 268; Vol. IV., 339; Vol. V........414
Bishop: Blood Pressure.........483
Bishop: Nose, Throat and Ear.555

PAGE.
Blakiston: Visiting List, 1905...343
Boston: Textbook of Clinical Diagnosis ...................480
Buck: Reference Handbook, Vol. VIII. ........................140
Cabot: Examination of the Blood ........................139
Chapin: Infant Feeding.........413
Cone: Man Pleases, Woman Charms ..................... 69
Croftan: Clinical Urinology.....331
Dana: Treatment of Nervous Diseases ....................464
Davis: Eye, Ear, Nose and Throat Nursing .............695
Davis: Obstetric and Gynecologic Nursing ................202
Davis: Obstetrics ..............481
Delafield; Pathological Anatomy, etc. .........................267
De Lee: Obstetrics for Nurses.. 69
Dudley: Principles and Practice of Gynecology................332
Dunham: Normal Histology....415
Durck: Pathologic Histology....554
Dwight: Toxicology............555
Edebohls: Surgical Treatment of Bright's Disease..............484
Edgar: Practice of Obstetrics...336
Einhorn: Diseases of the Intestines ........................ 67
Ferguson: Normal Histology...694
Fischer: Infant Feeding........ 69
Friedenwald: Diet in Health and Disease .....................482
Gazette Pocket Speller and Definer .........................342
Graffstrom: Mechano-Therapy..342
Griffith: Handbook of Surgery..487
Gould: American Yearbook, 1905.629
Gould: Diseases of the Eye....695
Haab: Atlas of Ophthalmology.693
Hall: Experimental Physiology.486
Hamilton: Railway Accidents and the Nervous System......269
Hare: Progressive Medicine, Sixth Series, Vol. II., 69; Vol. III., 340; Vol. IV., 626; Seventh Series, Vol. I............696
Hare: Textbook of Therapeutics.626
Hatcher: Textbook of Materia Medica ......................485
Head: Practical Medicine Yearbooks, Third Series, Vol. IV., Gynecology, 66; Vol. V., Obstetrics, 203; Vol. VI., General Medicine, 269; Vol. VIII., IX. and X., Materia Medica, Physiology, etc., Skin and Mental Diseases .....................624

PAGE.
Holland: Urine, Common Poisons, etc. .....338
Holmes: Appendicitis .....627
Hughes: Practice of Medicine. .697
Hyatt-Woolf: Optical Dictionary.342
Hyde: Diseases of the Skin.....338
Jackson: Ophthalmic Yearbook, 1903 .....696
Janeway: Study of Blood Pressure .....270
Judson: Growth and Deformities .....768
Kelly-Hurdon: Vermiform Appendix .....827
Kirke: Handbook of Physiology.485
Kraaft-Ebing: Textbook of Insanity .....621
Lea: Medical News Visiting List, 1905 .....343
Lenhartz: Clinical Microscopy and Chemistry..... 65
v. Leube: Medical Diagnosis....201
Lippincott: International Clinics, Fourteenth Series, Vol. I., 62; Vol. II., 141; Vol. III., 340; Vol. IV.....628
Lydston: Genitourinary and Sexual Diseases.....766
McFarland: Textbook of Pathology .....412
Magee: Surgery.....416
Medical Directory of N. Y., N. J. and Conn., 1904-5.....341
Morris: Materia Medica.....558
Moynihan: Surgical Treatment of Gallstones.....478
Muir: Materia Medica and Pharmacy ..... 68
Musser: Medical Diagnosis.....139
Nagel: Nervous and Mental Diseases .....416
Nancrede: Essentials of Anatomy .....557
v. Noorden: Metabolism and Nutrition, Part V.....201
Nothnagel: Encyclopedia of Medicine, Vol. VII., 63; Vol. VIII. .....137
Ott: Physiology..... 65
Pattee: Practical Dietetics.....770
Pearce: Nervous Diseases.....198
Penrose: Diseases of Women. . .487
Peterson-Haines: Legal Medicine and Toxicology, Vol. II.....330
Preble: Pneumonia, etc.....697
Pusey-Caldwell: Röentgen Rays.482
Pyle: Nose and Throat Diseases.267
Pyle: Personal Hygiene.....627
Reese: Vertebrate Embryology. .341
Reed: Diseases of Stomach, etc.479

PAGE.
REPORTS:
Michigan State Board of Health, 1902 .....558
N. Y. State Department of Health, 1902 .....625
Presbyterian Hospital, New York, Vol. VI.....271
Public Health and Marine Hospital Service, 1903....202
Public Health and Marine Hospital Service, 1904....624

Ricketts: Surgery of Heart and Lungs .....335
Ricketts: Surgery of the Prostate .....556
Robinson: Utero-Ovarian Artery.270
Rolleston: Diseases of the Liver, etc. .....478
Roosa-Davis: Diseases of Eye and Ear .....557
Sachse: How to Cook for the Sick, etc.....487
Saint Clair: Medical Latin.....629
Saxe: Examination of the Urine.266
Shaw: Nervous Diseases and Insanity .....556
Simon: Clinical Diagnosis..... 67
Simon: Textbook of Physiological Chemistry.....483
Spratling: Epilepsy and its Treatment .....199
Stoney: Materia Medical for Nurses .....341
Taylor: Genitourinary, Venereal Diseases and Syphilis.....265
Thompson: Graves's Disease....200
Thorington: How to Refract...557
Thornton: Pocket Formulary... 68

TRANSACTIONS:
American Medico-Psychological Association, 1903....133
American Association of Obstetricians and Gynecologists, 1903.....336
American Laryngological, Rhinological and Otological Society, 1903.....558
American Otological Society, 1904 .....630
American Gynecological Society, 1904.....697
American Surgical Association, 1904 .....698
American Laryngological Association, 1904.....769
Medical Society State of New York, 1904.....334

PAGE.

Southern Surgical and Gynecological Association, 1903.337

Treat: International Medical Annual, 1905....................769
Tuley: Epitome of Pediatrics... 64
Tyson: Bright's Disease and Diabetes ........................553
University of Pennsylvania: William Pepper Laboratory Reprints, No. 4.................488
Van Schaick: Regional Minor Surgery .....................488
Walker: Beauty through Hygiene ........................770
Wilcox: Fever Nursing........ 64
Whiting: Modern Mastoid Operation ........................692
Wolff: Essentials of Chemistry..486
Wood: Medical Record Visiting List, 1905.....................343

Renner, W. Scott, Treatment of Otitis Media ........................720
Rheumatism, Analgesic Salve for...172
Ricketts, M. B., Rupture of Gallbladder .............................596
Rinderpest, Investigation of by Koch.404
Rochester, DeLancey, Review of the Tuberculosis Problem.............701
Röntgen Rays, Pathological Action of ..............................540
Rowell, E. E., Considerations Relating to Convalescents..............792
Rubber Gloves, Substitute for....... 46
Ruggles, E. Wood, A. New Genito-urinary Table ..........515
E. Wood, Epidemic of Scabies ................280

SALINE Infusion in Enteric Fever 34
Salpingitis in a Child........657
Saint Bartholomew's Hospital, Incident at ..........................682
Sawers, F. H., Quarantine..........646
Scabies ............................173
Recent Epidemic of.........280
Septicemia .........................385
Sexton, J. C., Surgery of Nephritis.586
Shock, Hypodermic Injection in.....511
Simpson, Burton T., Athletics in Professional Colleges.............435
Shurly, E. L., Etiology of Appendicitis ..............................773
Snell, Albert C., Electro-Magnet in Eye Work ......................515

PAGE.

**Society Meetings:**

American Microscopical Society. 58
American Neurological Association .....................58, 128
American Electrotherapeutic Association ................ 59 826
American Medical Association... 60
American Association of Obstetricians and Gynecologists....128
American Medical Editors' Association ....................129
American Public Health Association ........................260
Amer. Antituberculosis League.552
American Academy of Ophthalmology and Oto-Laryngology.687
American Gynecological Society.688
Association of Military Surgeons of the U. S...............57, 195
Association of U. S. Pension Examining Surgeons............765
Buffalo Academy of Medicine, ........59, 192, 197, 259, 328, 411, 476, 552, 619, 687, 765, 827
Buffalo Ophthalmological Club..688
Canadian Medical Association..196
Canandaigua Medical Society...475
Elmira Academy of Medicine..476
Erie County Medical Association.................258, 411, 620
Esculapian Club.............. ........260, 476, 552, 619, 687
Harvard Medical Society of N. Y. ..........................328
Homeopathic Medical Society State of N. Y...............551
International Congress of Arts and Sciences .................130
International Society of Surgery.826
Lake Erie Medical Society...... 60
Lake Keuka Medical and Surgical Association ............... 60
Medical Association of Central New York..............195, 259
Medical Society, County of Chautauqua ...................58, 411
Medical Society County of Erie ..........................412, 475
Medical Society State of New York ...............196, 410, 474
Mississippi Valley Medical Association .............60, 197, 258
N. Y. and N. E. Association of Railway Surgeons............195
N. Y. State Medical Association ....259, 260, 476, 552, 619, 687
Pan-American Medical Congress (Fourth)....59, 129, 196, 257, 327
Physicians' Club................620

PAGE.
Physicians' League of Buffalo...826
Southern Surgical and Gynecological Association.......327, 475

**Society Proceedings:**

American Society of Clinical Surgery .....................287
Buffalo Academy of Medicine, Section on Medicine..........
297, 303, 455, 535, 648, 794, 797
Medical Association of Central New York ..................391
Medical Society, County of Erie
.............21, 526, 591, 804

Spitting Nuisance, Campaign against.549
Spleen, Not a Physiologic Necessity.322
State Cancer Laboratory Appropriation ............................823
Stephenson, Frank, Toxemia and Infections as Causes of Insanity....362
Sterility Due to the Man........... 30
Treatment of.............. 32
Streets, Education in Keeping Clean.325
Sutures, Emergency ................793
Syphilitic Reinfection...............187

TABLE, A New Genitourinary..517
Temple, Franklin Stuart, Conviction of.......................254
Temple, Franklin Stuart, to Pay Fine.684
Tetanus, Morphine and Eucain in...170
Thompson, F. D., Fibroid Complicating Gestation ...................452
Tonsillitis .........................286

**Topics of Public Interest:**

Agreement not to Practise in Given Locality, Validity of....321
Army Medical Service, Examination for..................43, 542
Canal Zone Sanitation, Report of Dr. Charles A. L. Reed on....599
Christian Science, Death from..401
Civil Service Examinations.542, 611
Council Meeting, N. Y. S. M. A.176
Crusade, A Modern............245
Dentists, Municipal.............671
Insane, N. Y. State Hospitals for.319
Japanese Medical Department...316
Library, The State Medical.....316
Milk Bottles, Paper............669
N. Y. S. M. A., The............178
Optometry Campaign, Address of Chairman Van Fleet in......399

PAGE.
Osteopathic Bill, Defeat of in New York and Pennsylvania.755
Osteopathic Bill, Physicians Oppose ........................659
Panama Canal Zone, Medical Service of ...................317
Panama, Fever at..............759
Pneumonia Commission, N. Y. Board of Health.............248
Roosa, Dr., Asks the Title Doctor of Medicine be Safeguarded ..........................668
Substitution again Punished.... 45
Tuberculosis, Crusade Against..247
Unification Failure as Others See it ..........................315
Unite, Why We Did Not......173
Vaccination in Schools.........245

Treatment by Means Other Than Drugs ............................802
Tuberculosis, Disinfection of Houses.255
History of............381
Observations on.......314
Problem, Review of..701
Typhoid Diagnostician, Ficker's..... 36
Fever, Treatment of Hemorrhage in............... 34
Fever, Turpentine in...... 36
Perforations .............. 33

ULCER of the Leg, Treatment of.117
Unification Scheme, Explanatory of Failure of..................189
University Extension ..............822
Extension, Committees on.762
State of New York Convocation of............ 53
Lectures ................255

**University and Hospital Bulletin:**

Alumni Association, Meeting of.727
American Society of Clinical Surgery ....................287
American Society of Clinical Surgery, Meeting at Buffalo..234
Aneurism of Left Carotid, Park.235
Annual Theater Party..........382
Banquet, Speeches at...........732
Bulletin Notes.........236, 237, 238
Changes in Permanent Faculty.158
Commencement Ceremonies ....729
Crockett, Dr. Montgomery A., Year's Leave of Absence......466
Cutaneous Growths, Degeneration of ......................159

Page.
Department of Anatomy, U. of B. ....462
Fibroma of Temporo-Frontal Region, Park ....234
Gallbladder, Removal of........383
Gallstone Cases, Unusual.......161
Gastroenterostomy and Malignant Growth of Liver........291
Hernia, Multiple ....525
Hernia of Bladder with Complications ....524
Hernia, Strangulated Umbilical..384
Hydatiform Mole, Hayd........526
Hydrocephalus, Arachno-Abdominal Drainage for.............160
Intestinal Obstruction, Operation by Dr. Hayd.................160
Introductory ....157
Jaw, Resection of for Ankylosis.384
King, James E., Resignation of at Hospital....................466
Liver, Hydatid Cyst of.........384
Mann, E. C., Appointment of at Hospital ....466
Mercy Hospital, First Operation at, Dowd ....239
Opening of Medical Department.158
Pathological Department........382
Pelvis, Repair of Fractured.....383
Simpson, Burton T., To Teach Embryology ....466
Teaching of Physiology, U. of B. ....521
Thrombosis of Left Femoral Artery, Eugene A. Smith.......235
Thyroidectomy, Crile's Procedure in, Park................291
Tibia, Unusual Fracture of.....382
University Athletics ....385
University of Buffalo, Commencement Week, 1905.............727

Page.
Vertebras, Resection of Ninth and Tenth Dorsal, Parmenter.292

Uremic Convulsions................395
Uric Acid, Determination of in Urine.166
Urinalysis in Pregnancy............ 31
Urine in Pregnancy................. 30
Causes of Black.............167
Urotropin and Helmitol.............310
in General Paralysis.....597
Uterine Cancer, Pregnancy and Forceps Delivery in................. 28

VAGINAL Fixation and Ventrosuspension, Pregnancy After..240
Van der Beek, C. A., Care of the Insane ....275
Veratum Viride in Surgery......... 27
Vomiting, Recurrent................655
Vulvæ Pruritus ....279

WEICHERS, Conviction of.....405
Wey, Hamilton D., What Medicine is Accomplishing Today.491
Williams, Herbert U., Notes of Travel in Mexico.....................377
Wilson, Nelson W., Chinese Medicine and Surgery in New York........153
Women, Circumcision of............107
Unclassified Troubles of...241
Wounds of the Extremities, Treatment of Infected................284

YEAST as an Antigonorrheic....163

